# Effects of Antiviral Therapy on the Recurrence of Hepatocellular Carcinoma After Curative Resection or Liver Transplantation

**DOI:** 10.5812/hepatmon.6031

**Published:** 2012-10-20

**Authors:** Yan Du, Tong Su, Yibo Ding, Guangwen Cao

**Affiliations:** 1 Department of Epidemiology, Shanghai Key Laboratory of Medical Biodefense, Second Military Medical University, Shanghai, China

**Keywords:** Carcinoma, Hepatocellular, Surgical Procedures, Operative, Recurrence, Survival

## Abstract

**Context:**

Hepatocellular carcinoma (HCC) is a fatal disease. Chronic hepatitis B virus (HBV) and/or hepatitis C virus (HCV) infection is the major cause of HCC. High viral replication rate and related hepatic/systematic inflammation are the major risk factors in HCC recurrence after hepatectomy or liver transplantation.

**Evidence Acquisition:**

Some of the carcinogenesis-related HBV mutations are also associated with poor prognosis for HCC patients. Antiviral therapy is an option for improving HCC prognosis after surgery. In case of HBV-associated HCC, treatment with interferon and nucleos(t)ide analogues (NAs), especially interferon, is effective in improving the prognosis. However, long-term use of NAs increases the possibility of developing drug-resistant viral mutations such as the HBV rtA181T/sW172 mutation, which increases the risk of HCC recurrence.

**Results:**

In cases of HCV-associated HCC, standard interferon with or without ribavirin therapy is effective in improving the prognosis of HCV-associated HCC; however, some HCV mutations, such as the amino acid substitution M91L, are associated with treatment failure and a poor prognosis. Therapeutic efficacy needs to be confirmed using largescale, randomized, placebo-controlled clinical trials.

**Conclusions:**

Surveillance of viral mutations during antiviral treatment and a better understanding of the associations of HCC recurrence with viral load, inflammation-associated signaling, and environmental factors can aid the development of more effective strategies for the prevention of HCC recurrence after surgery.

## 1. Context

Hepatocellular carcinoma (HCC) is the sixth most common malignancy and the third leading cause of cancer death worldwide (1). Chronic infection with hepatitis B virus (HBV) and hepatitis C virus (HCV) accounts for about 75–80% of HCC cases worldwide (2). In Asia and Africa, where HCC is endemic, chronic HBV infection is the predominant risk factor, while in the western countries, HCV infection is one of the major risk factors. HCC is a fatal disease. Currently, orthotopic liver transplantation (OLT) and surgical resection are the only curative treatments. OLT has excellent outcomes in patients meeting the Milan criteria (single nodule of ≤ 5 cm or 2 or 3 nodules of ≤ 3 cm), with a 5-year survival rate of 70%. Nevertheless, because of the strict selection criteria and high costs associated with the therapy, it can be offered to only a small fraction of the affected patients (3). Therefore, surgical resection is the main curative treatment for noncirrhosis patients and cirrhosis patients with well-preserved liver function. However, it is reported that up to 70% of the patients show relapse within 5 years after curative resection (4). The high rate of recurrence is a major obstacle to improving prognosis. Early recurrence (within 2 years) is mainly related to metastasis and dissemination of primary HCC, whereas late recurrence (after ≥ 2 years) mostly results from de novo tumors arising because of the “field effect” in the diseased liver and is closely associated with high viral loads and hepatic inflammatory activity (5, 6). Therefore, antiviral and anti-inflammatory therapies before and after curative treatment may be crucial in preventing HCC recurrence and in improving survival. Current approved medications for chronic hepatitis B (CHB) treatment are interferon-α (IFNα) and nucleos(t)ide analogues (NAs), including lamivudine (LAM), entecavir (ETV), tenofovir disoproxil fumarate (TDF), adefovir dipivoxil (ADV), and telbivudine (TBV) (7). Conventional treatment with IFNa and the pegylated, long-acting formulation (PEG-IFNa) in combination with the guanosine analog ribavirin (RBV), are considered a standard modality for chronic hepatitis C treatment (8). There are no anti-inflammatory drugs available for the prevention of HCC recurrence after surgery, but antiviral treatment and antioxidants can decrease liver inflammation (9). In this review article, we have re-evaluated the reported effects of antiviral treatments on the occurrence of HCC after surgical treatment, and we have pointed out existing problems in current studies.

## 2. Evidence Acquisition

### 2.1. Why do HCC Patients Need Postoperative Antiviral Treatment and What are the Characteristics of These Patients?

High viral loads in the serum or liver and hepatitis B e antigen (HBeAg) seropositivity indicate a high viral replication rate. The presence of HBeAg either before or after curative treatment for HCC is significantly associated with early recurrence and poor survival (10-12). Serum hepatitis B core-related antigen (HBcrAg), consisting of HBeAg, hepatitis B core antigen, and a 22-kDa precore protein coded with HBV precore/core gene, could be a surrogate marker for the intrahepatic covalently closed circular DNA (cccDNA) pool. A high serum level of HBcrAg is an independent factor in HCC recurrence (13). The severity of hepatic inflammation, which is well correlated with viral serostatus, may also be a factor that affects intrahepatic recurrence, which is more likely to originate from metachronous carcinogenesis (14). High levels of HBV DNA in peritumoral liver tissues of HCC patients independently predicted poor disease free survival (DFS) and overall survival (OS) after surgical resection (15). Sustained low hepatitis B viral load (< 10^4^ copies/mL) is significantly associated with improved long-term recurrence-free survival and OS (16). In addition, HBV viral load is one of the main prognostic factors for local recurrence after complete radiofrequency ablation (RFA) of small HBV-related HCC (12). Thus far, there is not much information on the association between HCV RNA concentration and HCC recurrence after surgery. The available data has shown that HCV concentration is an independent prognostic factor for OS and recurrence (17, 18). These data indicate that high rates of viral replication are positively associated with a high risk of HCC recurrence after surgery. Chronic inflammation supported by chronic HBV or HCV infection orchestrates a tumor-friendly microenvironment that is essential for carcinogenesis and metastasis. Chronic inflammation and high viral replication rate are important predictors of adverse outcome after HCC curative treatments. Chronic inflammation plays a crucial role in cancer initiation and promotion. Abnormal inflammation, including aberrant production of pro-inflammatory mediators and increased expression of oncogenes, matrix metalloproteinases, and pro-inflammatory transcription factors such as nuclear factor kappa-light-chain-enhancer of activated B cells (NF-κB), signal transducers and activators of transcription 3 (STAT3), activating protein-1 (AP-1), and hypoxia-induced factor-1α (HIF-1α) can activate genes mediating tumor cell proliferation, survival, invasion, and angiogenesis (19). High viral replication rates are closely related to hepatic inflammation. Many studies have provided evidence that inflammation-related host factors can predict HCC recurrence and survival after surgical resection or liver transplantation (20-22). All these data support the finding that persistent viral infection-associated inflammation plays an active role in the recurrence of HCC. Inflammation contributes to the formation of HBV mutations and the mutants can in turn facilitate HCC occurrence and progression. One of the common mechanisms of HBV mutagenesis to escape immune clearance is the reduction of CD8^+^ T cell epitopes. Some of the HBV mutations selected by a compromised immune system during HBV hepatocarcinogenesis are significantly associated with an increased risk of HCC (23-25). C1653T, T1753V, A1762T/G1764A, T1674C/G, C1766T/T1768A, T53C, preS2 start codon mutation, preS1 deletion, C2964A, A2962G, C3116T, C7A, and their combinations are HBV mutations that are significantly associated with an increased risk of HCC occurrence (25-27). PreS deletion is the most common mutation in the preS region. The preS mutations may be generated during the progression of CHB, particularly in IFN-treated patients (28). The preS deletion also affects viral replication by decreasing the expression of surface proteins, which leads to intracellular accumulation of HBV envelope proteins and viral particles, formation of ground-glass hepatocytes, endoplasmic reticulum stress, and oxidative DNA damage (29). All these changes eventually result in hepatocarcinogenesis. In peritumoral tissues, a preS deletion at nt.107-141 and preS2 mutations are independently associated with poor DFS and OS after surgery (23, 30). A1762T/G1764A in liver tissue can independently predict postoperative survival (15). HCV is hypervariable in a region coding for envelope proteins and escapes immune surveillance. It has been reported that 2 amino acid substitutions in the core region of HCV-1b, Q70R and M91L, are significantly associated with resistance to the standard IFNa plus RBV therapy and an increased risk of HCC (31). Moreover, M91L is significantly associated with recurrence and poor survival in HCC patients after surgery (32). Currently, there are no data showing that the viruses with HCC- or HCC prognosis-associated mutations are still sensitive to IFN and/or NA treatments. We, therefore, suggest that HCC patients who need postoperative antiviral treatments are those who (1) have a high HBV DNA level (> 104 copies/ml) at the time of surgery (2); are seropositive for HBeAg or have a high serum level of HBcrAg (3); are infected with HBV with the HCC-or HCC prognosis-associated mutations (4); have recurrent HCV after OLT/hepatectomy or HCV with HCC prognosis-associated mutations (5); have high Ishak hepatic inflammation score (> 6) or abnormal alanine aminotransferase (ALT); and (6) have over-expression of inflammation-related molecules in HCC specimens or peritumoral liver tissues. Furthermore, since HCC curative resection may reactivate HBV replication (33), HCC patients with a high level of HBV reactivation within 3 months after surgery should also be considered for antiviral treatment.

### 2.2. Antiviral Treatment Improves HCC Prognosis

#### 2.2.1. Effects of IFN on HBV- or HCV-Related HCC Survival and Recurrence

A meta-analysis conducted by Breitenstein et al. (34) pooled data from 7 randomized clinical trials (RCTs) (35-41) between January 1998 and October 2007 and concluded that IFNa had a significant beneficial effect on both survival and tumor recurrence. Two additional meta-analyses published in 2010 (42, 43), including RCTs and non-randomized controlled trails (NRCTs), reported similar results (44-51). Other recent studies have also supported the role of IFN treatment in preventing early recurrence and improving survival after curative treatment of HCC (52-54). However, these studies do not separate HBV-related HCC from HCV-related HCC. Since HBV and HCV have distinctive characteristics and therefore different regimens (i.e., IFN/NAs for HBV-related HCC *vs*. IFN/RBV for HCV-related HCC) after curative surgery, we summarized the results of HBV-related HCC RCTs and NRCTs in Table 1. For HCV patients, IFNa and PEG-IFN can achieve sustained virologic response (SVR), seronegative for HCV RNA throughout the 6-month post-treatment follow-up period. The patients who achieve SVR following treatment with IFN and RBV usually have a good prognosis; however, in those who do not respond to initial antiviral therapy, maintaining IFN therapy may not decrease HCC recurrence. These results are summarized in Table 2. HCC recurrence rates and related deaths were significantly lower in patients who received post-OLT IFN therapy for recurrent HCV (58). Since high viral load is frequently associated with late recurrence of HCC after surgery, antiviral treatment should be solely effective for the prevention of late recurrence. However, it can also efficiently prevent early recurrence of HCC after surgery (52-54). IFN is effective in preventing both early and late recurrence of HCC, possibly due to its effects on angiogenesis, Wnt/β-catenin pathways, and immune modulation. Vascular invasion (microscopic vascular invasion or macroscopic venous invasion) is associated with early HCC recurrence (6). IFNα inhibits metastasis and early recurrence of human HCC after curative resection, which is possibly mediated by anti-angiogenesis through down-regulation of expression of vascular endothelial growth factor (VEGF) (55-57). The expression of HBx in hepatocytes activates Wnt/β-catenin signaling, and Wnt pathway activation induced by β-catenin mutations is associated with a poor prognosis (22, 58, 59). PEG-IFN targets Wnt signaling by inducing nuclear export of β-catenin, and thus affects the recurrence of HCC (60). It is believed that the principal mechanisms of IFN in prevention of HCC recurrence in patients with viral hepatitis are the suppression of HBV and HCV replication, inhibition of inflammatory signaling, and tumoricidal effect (55, 57). IFN treatment has adverse effects, including flu-like symptoms, fatigue, neutropenia, thrombocytopenia, depression, bone marrow suppression, and unmasking or exacerbation of autoimmune illnesses. These are generally tolerable but may require dose modification and premature withdrawal from the treatment. In addition, antibodies to recombinant IFN, which might be generated during long-term treatment, may limit its biological effects.

**Table 1:** Studies of Effects of IFN on HBV-Related HCC Survival and Recurrence After Surgical Resection

	Patients	Therapy	Survival (OS, DFS, RFS)	Recurrence
**RCT**				
Lin, et al. (2004) (38)	30 patients after non-surgical treatment (transarterial chemoembolization or percutaneous acetic acid injection) of HCV- or HBV - related HCC nodules	IFN-α intramuscular injection. Treatment group A: 3 MIU × 3/week × 24 months. Treatment group B: 3 MIU × 10/month × 6 months, then 3 MIU × 10/3month ×18 months	Not analyzed	The HCC recurrence rate among untreated patients was 40% at 1 year, 70% at two years, and 90% at 4 years and was higher than the rates among patients treated with IFN-α (25% at 1 year, 30% at two years, and 47% at four years)
Sun, et al. (2006) (39)	236 patients after curative resection of HBV-related HCC	IFN-α intramuscular injection, 3 MIU × 2/week × 2 weeks, then 5 MIU × 3/week × 18 months	Treated *vs.* control: Median OS, 63.8/38.8 months (P = 0.0003); Median DFS, 31.2/17.7 months, P = 0.42	IFN-α treatment improved the OS, probably by postponing recurrence.
Lo, et al. (2007) (41)	80 patients after curative resection of predominantly HBV -related HCC	IFN-α2b subcutaneous injection, 10 MIU× 3/week × 16 weeks	Adjusted RR of death for IFN treatment was 0.42 (95%CI: 0.17–1.05; P = 0.063)	No significant difference in the overall DFS
**NRCT**				
Someya, et al. (2006)(48)	80 patients with HBV -positive cirrhosis and HCC underwent curative treatment (surgical resection or sufficient ablation) for HCC	Intermittent IFN-α injections, 2-3/week × 6 months or longer.	Not analyzed	In the subgroup of abnormal AST, HCC recurrence rates in the IFN group were significantly lower than the non-IFN group (P = 0.0139).
Qu, et al. (2010) (52)	568 HBV-related HCC patients underwent curative resection. A median observation period of 53.3 months	IFN -α1b intramuscular injection, 3 MIU × 2/week × 2 weeks, and then 5 MIU × 3/week × 18 months.	Postoperative IFN-a therapy was an independent factor for OS. No significant difference in DFS rates	Postoperative IFN-a therapy significantly reduced early recurrence.
Chan, et al. (2011) (53)	136 HBV -related HCC received hepatectomy	Antiviral therapy after hepatectomy	Antiviral treatment conferred a significant survival benefit in stages I and II tumors or HCC without major venous invasion	Not analyzed

Abbreviations: AST, Aspartate aminotransferase; DFS, Disease free survival; HBV, Hepatitis B virus; HCC, Hepatocellular carcinoma; IFN, Interferon; MIU, Million international units; NRCT, Non-randomized controlled trail; OS, Overall survival; RCT, Randomized clinical trial; RFS, Recurrence free survival.

**Table 2:** Effects of IFN on HCV-Related HCC Survival and Recurrence After Surgical Resection

	Patients	Therapy	Survival (OS, DFS, RFS)	Recurrence
**RCT**				
Ikeda, *et al.*(2000) (35)	20 patients with HCV infection had received curative resection.	IFN-β injection, 6 MIU× 2/week × 36 months.	Not analyzed	Treated *vs. *controls: 2 year cumulative recurrence rates: 0% *vs. *100%, *P* = 0.0004
Kubo*, et al.*(2002) (36)	30 males with HCV infection and curative surgical resection of a single HCC tumor.	IFN-α intramuscular injection, 6 MIU × 7/week × 2 weeks, then 6 MIU × 3/week × 14 weeks, then 6 MIU × 2/week × 88 weeks.	The cumulative survival rate was higher in the IFN group than in the control group (*P* = 0.041).	Treated *vs.* controls: 4 year intrahepatic recurrence (9/13), *P* = 0.055
Shiratori,* et al.* (2003) (37)	74 patients with compensated cirrhosis, three or fewer nodules of HCC, and low HCV RNA loads after complete ablation of the lesions.	IFN-α intramuscular injection, 6 MIU × 3/week × 48 weeks	The survival rate was higher in the IFN group than in the control group	Similar in 1st recurrence rate; Lower 2nd or 3rd recurrence rates in IFN group than the control group
Mazzaferro*, et al.*(2006) (40)	150 patients after curative resection of HCV-related (n = 80) or HCV and HBV-related (n = 70) HCC.	IFN-α subcutaneous injection, 3 MIU × 3/week × 48 weeks	Treated *vs.* control: 45 months of median follow-up; RFS: 24.3% *vs. *5.8%, *P* = 0.49	IFN did not affect overall prevention of HCC recurrence, but it may reduce late recurrence in HCV-free patients receiving effective treatment (*P *= 0.04).
**NRCT**				
Suou*, et al.*(2001) (44)	40 patients after curative treatment of small HCV -HCC (solitary, diameter ≤ 3 cm), ≤ 70 years.	IFN α or IFN-α2b intramuscular injection, 6 MIU × 7/week × 2 weeks, then 6 MIU × 3/week × 22 weeks.	The cumulative survival rate was significantly longer in the IFN group compared with the control group (*P* < 0.01).	IFN α therapy after the curative treatment of small HCC with HCV can inhibit intrahepatic recurrence and improve the prognosis of HCV-related HCC
Hung*, et al.*(2005) (45)	40 patients with 3 or fewer nodules of HCV-related HCC a who received percutaneous tumor ablation and/or transcatheter arterial embolization.	IFN-α2b subcutaneous injection, 3-5 MIU a × 2/week × 24-48 weeks, with the combination of oral ribavirin 1000-1200 mg/day for 24-48 weeks	Survival in sustained responders was better than in non-responders and control patients (*P* = 0.0691, 0.0554, respectively)	No significant difference in the incidence of local recurrence in sustained responders; the 2nd recurrence-free interval in the sustained responders was significantly longer than non-responders and control group
Sakaguchi,* et al.*(2005) (46)	57 patients with HCV -related HCC underwent radical RFA therapy.	IFN-α2b intramuscular injection, 3 MIU × 2/week for as long as possible	There was no difference in the cumulative survival rates between the IFN group and the control group (*P* = 0.25)	The median tumor-free period was longer in the IFN group than the control. The cumulative recurrence rate in the IFN group was lower than the control during the first 3 years; however, the recurrence rate in the IFN group increased over 3 years
Akamatsu,* et al.*(2006) (47)	643 HCV-related HCC patients who underwent curative treatment (surgical resection or ablation).	IFN-α injection; 3-6 MIU × 3/week × 24-48 weeks	IFN therapies were significantly associated with prolonged survival when SVR was achieved	RFS did not differ significantly.
Kudo, *et al.* (2007) (49)	Matched case-control study: 127 HCC (tumor diameter ≤ 3cm, number of tumors ≤ 3) curatively treated by RFA	IFN-α2b 3 MIU × 2/week, or PEG -IFN-α2a 90μg × 1/(1-2)week	Maintenance *vs.* control: 5 year survival rate, 83% *vs.* 66%. IFN a maintenance therapy was an independent risk factor for survival.	Cumulative 1st, 2nd, and 3rd recurrence rates were significantly reduced in the IFN maintenance group compared with the control group
Jeong,* et al*. (2007) (50)	84 patients underwent curative treatment for HCV-related HCC	IFN a -α intramuscular injection, 6 MIU × 7/week × 2 weeks, then 6 MIU × 3/week × 22 weeks	Survival rate in the SVR group was significantly better than that in the non-IFN group	Initial recurrence rate did not differ. IFN group showed a lower rate than the non-IFN group for 2nd recurrence
Jeong,* et al.*(2007) (50)	32 patients with HCV-related compensated cirrhosis after curative HCC treatment.	IFN-α intramuscular injection, 3 MIU × 3/week × 48 weeks or longer.	The cumulative survival rate was not significantly different between the two groups for first 4 years	The cumulative rate of HCC recurrence was not significantly different between the IFN group and the non-IFN group.
Ishikawa,* et al.* (2011) (54)	54 patients with initial HCV -associated Stage I/II HCC underwent curative treatment.	PEG-IFN-α2b with the combination of ribavirin	PEG-IFN a-2b/ Ribavirin therapy following HCC treatment shows promise for improving the prognosis of HCC	Not analyzed

Abbreviations: DFS, Disease free survival; HCC, Hepatocellular carcinoma; HCV, Hepatitis C virus; IFN, Interferon; MIU, Million international units; NRCT, Non-randomized controlled trail; OS, Overall survival; RCT,Randomized clinical trial; RFA, Radio frequency ablation; RFS, Recurrence free survival; SVR, Sustained virologic response.

#### 2.2.2. Effects of NAs on HCC Survival and Recurrence

HBV-positive patients require both sufficient antiviral therapy with NAs and hepatitis B immune globulins (HBIG) after successful liver transplantation to effectively prevent recurrence (61). The introduction of HBIG treatment greatly reduces HBV recurrence after HBV-related OLT. Even thought there is no current consensus on the optimal HBIG dosage and duration, it is widely agreed that HBIG plasma titers should be maintained at a level of least 100 IU/L during long-term therapy (62). The aims of NA treatment are to inhibit HBV DNA replication, normalize ALT levels, and maintain liver function. NAs target HBV DNA polymerase. Short-term treatment with NAs (< 6 months) can prevent post OLT HBV recurrence (63). Since HBcrAg is a predictor of post-treatment recurrence of HCC, suppression of serum HBcrAg and cccDNA by NAs may be important to prevent HCC recurrence (13). LAM is the first NA to treat CHB. It can inhibit viral replication, improve liver function, and induce histological improvement of fibrosis (64). However, it has shortcomings, including development of drug resistant strains and attenuation of HBV suppression and other serious clinical challenges (65). The current standard of therapy is either TDF or ETV. In patients mono-infected with HBV, TDF seems to have a low drug resistance rate and good tolerability, as well as few clinically significant side effects. ETV has been found to be a superior antiviral agent with a high genetic barrier. Therapy with ETV is more likely to induce a significant decline in viral loads in both HBeAg-positive and -negative treatment-naïve patients (7). Notably, treatment with NAs does not reduce early recurrence, but it promotes postoperative viral clearance and improves liver function. Several studies have compared the prognosis of HCC patients with and without NA treatments (66-71). Although most of the studies had small sample sizes and relatively short follow-up times, in general the NA treatments exhibited a potential beneficial effect in preventing HCC recurrence and improving survival after curative treatments (Table 3). Long-term usage of NAs is required to effectively inhibit HBV and maintain a low HBV load; however, this strategy leads to a major challenge in HCC management-drug resistance.

**Table 3:** Effects of NA Therapy on HCC Survival and Recurrence After Curative Treatment

	Patients	Therapy	Survival (OS, DFS)	Recurrence Rate
**Surgical Resection (including RFA)**				
**Piao, *et al.* (2005) (66)**	70 HCC patients completed HCC therapy (local ablation, trans-arterial chemoembolization, or surgery)	LAM: 100 mg/day orally for more than 24 months	There was no significant difference in the survivals between the two groups, but LAM treatment was associated with low cumulative rate of death due to liver failure (P = 0.043)	No difference was found between the treatment group and the control group (14/30 and 26/40)
**Kuzuya, *et al.* (2007) (67)**	49 HCC patients who underwent hepatic resection or RFA for initial HCC treatment.	LAM: 100 mg/day	The cumulative survivals of patients in the treatment group tended to be higher than those in the control (P = 0.063)	Cumulative recurrence rates of HCC did not significantly differ between the two groups (P = 0.622)
**Kubo, *et al.* (2007) (68)**	24 patients who had high serum concentrations of HBV DNA	LAM: 100 mg/day	Tumor-free survival rate was significantly higher in the treatment than the control group (P = 0.0086)	Not analyzed
**Yoshida, *et al. *(2008) (69)**	104 HCC patients underwent RFA treatment.	LAM: 100 mg/day	Overall survival did not differ between the two groups	Recurrence-free survival did not differ between the two groups
**Li, *et al.* (2010) (70)**	79 HCC patients underwent curative resection, a median follow-up of 12 months.	LAM with or without adefovir dipivoxil	OS was improved for those patients with postoperative antiviral therapy	No significant difference in recurrence rate between the treatment group and the control group (76.7% and 91.7%)
**Liver Transplantation**				
**Zimmerman, *et al.* (2007) (71)**	101 patients underwent OLT for end-stage liver disease secondary to HBV with concomitant HCC	LAM: 150 mg/day. HBIG: before 1998, 10,000U, iv, then 10,000U/d × 7 days, then 10,000 U/month. After 1998, 10,000U, iv, then 2000 U/d × 6 days, then 1560 U im	Patients treated with combination prophylaxis had a significantly lower mortality than those without	AFP > 500 ng/mL, presence of vascular invasion, HBV recurrence, and combination prophylaxis were independent predictors of HCC RFS

Abbreviations: DFS, Disease free survival; HCC, Hepatocellular carcinoma; HCV, Hepatitis C virus; IFN, Interferon; MIU, Million international units; NRCT, Non-randomized controlled trail; OS, Overall survival; RCT, Randomized clinical trial; RFA, Radio frequency ablation; RFS, Recurrence free survival; SVR, Sustained virologic response

#### 2.2.3. Drug-resistant Viral Mutations Limit NA Therapeutic Effect and May Also Promote Hepatocarcinogenesis

**Figure 1:**
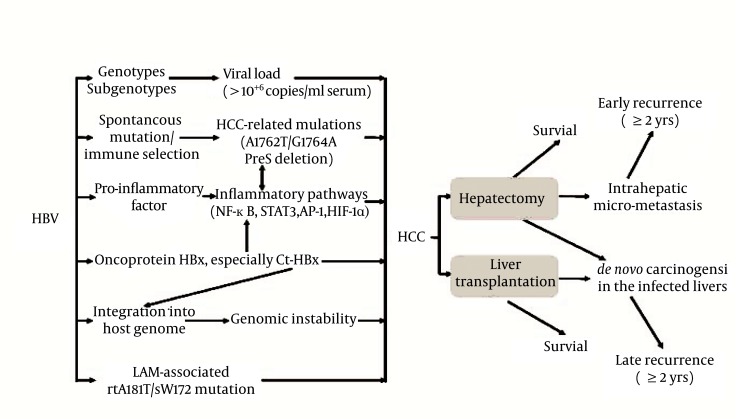
Major Events in HBV Hepatocarcinogenesis and HBV-Related HCC Prognosis HBIG, Hepatitis B Immune Globulin; HBV, Hepatitis B virus; HCC, Hepatocellular Carcinoma; IFN, Interferon; NA, Nucleos (t)ide Analogue

**Figure 2:**
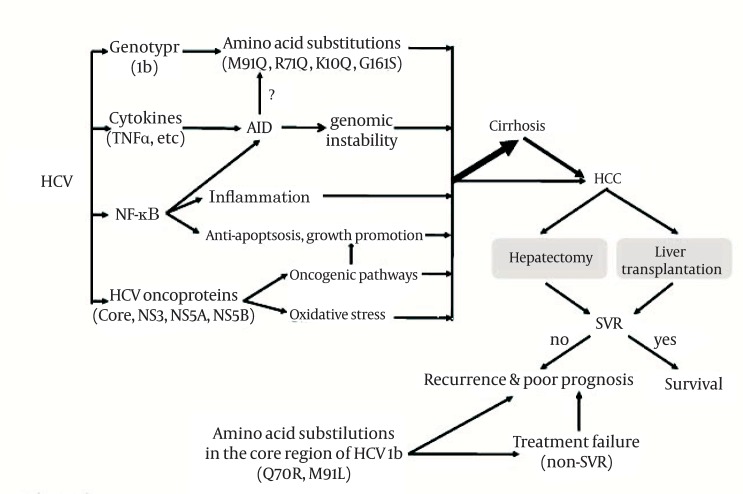
Major Events in HCV Hepatocarcinogenesis and HCV-Related HCC Prognosis HCC, Hepatocellular Carcinoma; HCV, hepatitis C Virus; SVR, Sustained Virologic Response

Long-term use of NAs may generate drug-resistant viral mutations. The most frequently used antiviral therapy for post-transplantation recurrence of HBV infection is LAM, but this drug is associated with a high resistance rate due to tyrosine-methionine-aspartate-aspartate (YMDD) mutants (72). The YMDD mutants may arise under immunosuppression, and emerge after 9-10 months of LAM therapy. The most frequently encountered LAM-resistant mutation at the catalytic YMDD motif is rtM204V/I (73-75, 78-80). The frequency of the mutation increases over the duration of LAM therapy year by year, up to almost 70% after 5 years (65). In terms of the current standard therapy, resistance to ETV is rare in treatment-naïve patients. However, in the presence of rtM204I/V mutations, ETV resistance arose with the coexistence of rtI169T, rtL180M, rtT184A/F/G/I/L/S, rtS202G/I, or rtM250V mutations (76, 77). As for other NAs, the rtN236T mutation is associated with ADV resistance (76, 78). The major TBV resistant mutant is rtM204I (79). Another mutant, rtA181T, may arise during prolonged LAM therapy, conferring cross resistance to ADV. Importantly, since the HBV S and polymerase genes overlap with each other, a great proportion of patients with the rtA181T mutation also carry the SW172 nonsense mutation, resulting in truncation of the preS/S reading frames, which significantly increases the risk of HCC during subsequent courses of NA therapy (80). Drug-resistant viral mutations generated during the long course of NA treatment are becoming one of the major risk factors of poorer HCC prognosis. The important events in HBV- or HCV-induced hepatocarcinogenesis and prognosis and the antiviral treatments for the prevention of HCC recurrence after surgical treatment are summarized in Figure 1 and Figure 2, respectively. 

## 3. Results

### 3.1. Regimen of Antiviral Therapy Suitable for the Prevention of HCC Recurrence

So far, there is no consensus on the standard regimen, such as drug combination, dosage, and optimal time of initiation of therapy, to achieve the best prognosis for HCC after curative treatment. The current practice is largely experience-based, and most results, especially for NAs, are from NRCTs. For IFN, previous studies have indicated that there was no difference between intermittent and continuous treatment strategies. The usual dosage is 3-6 million international units (MIU), with some with a larger dosage of 10 MIU, subcutaneously or intramuscularly. It is usually administrated 2-3 times per week, and needs to last for more than 6 months. It is also a common practice to add RBV to the IFN treatment regimen for HCV-related HCC. IFN has a short half-life in the circulation and needs frequent administration, thus can produce severe side effects. As a result, PEG-IFN has recently been prescribed more often, with a dosage ranging from 90 to 180 μg per week. As for NAs, LAM is an often-used agent, with a common dosage of 100 mg/day, sometimes in combination with another NA, such as ADV, to reduce the possibility of developing drug-resistance. Results from NRCTs show that NAs are effective in improving survival. However, it is difficult to make any recommendation based on these data to guide clinical practice. No definite conclusions could be drawn without creditable evidence from RCTs. International collaborations are needed to conduct large multi-centered RCTs in different populations in order to evaluate the most effective combination and administration of these therapeutic agents.

## 4. Conclusions

### 4.1. Summary and Suggestions

HBV and HCV related HCC cause a huge public health burden, especially in HBV endemic areas. Only a small proportion of HCC patients are eligible for curative treatment, namely surgical resection or OLT. Further, survival after the curative treatment is not optimal. High viral replication rates, viral mutations, and infection-associated inflammation are major factors associated with poor outcomes after surgery. Antiviral treatment is therefore an optimal option to prevent HCC recurrence and improve survival. IFN and NAs are currently the major antiviral agents in use. Use of antiviral agents not only inhibits virus replication and re-activation, but also decreases hepatic inflammation and can facilitate further treatment. IFN and NAs, especially IFN, have been proven to be effective in improving HCC prognosis. However, large multicenter RCTs are necessary to determine the most effective regimen of these antivirals in improving the HCC prognosis after surgery. The complex interactions among viral factors, host immunity, and environmental determinants may influence HCC recurrence and survival. However, the underlying mechanisms of this multi-way network have not yet been fully elucidated. A better understanding of the relationships among these factors can aid in developing advanced treatment strategies and improving the life quality of HCC patients. Future research should focus on the roles of viral factors, inflammation-related signaling molecules, and possible environmental factors on HCC occurrence and the effect of antiviral treatments on HCC - or HCC prognosis-associated viral mutants. A systematic scientific approach should be adopted to direct further studies.
